# The impact of different targeted temperatures on out-of-hospital cardiac arrest outcomes in patients receiving extracorporeal membrane oxygenation: a nationwide cohort study

**DOI:** 10.1186/s13054-022-04256-x

**Published:** 2022-12-08

**Authors:** Makoto Watanabe, Tasuku Matsuyama, Yuki Miyamoto, Tetsuhisa Kitamura, Sho Komukai, Bon Ohta

**Affiliations:** 1 Department of Emergency Medicine, Kyoto Prefectural University of Medicine, Kamigyo-ku, Kyoto, 602-8566 Japan; 2 Division of Environmental Medicine and Population Sciences, Department of Social and Environmental Medicine, Graduate School of Medicine, Osaka University, 2-2 Yamadaoka Suita, Osaka, 565-0871 Japan; 3 Division of Biomedical Statistics Department of Integrated Medicine, Graduate School of Medicine, Osaka University, Osaka, Japan

**Keywords:** ECMO, Targeted temperature management, Out-of-hospital cardiac arrest

## Abstract

**Background:**

Targeted temperature management (TTM) is recommended in the management of out-of-hospital cardiac arrest (OHCA) when coma persists after the return of spontaneous circulation. In the setting of extracorporeal membrane oxygenation (ECMO) for OHCA patients, TTM is associated with good neurological outcomes and is recommended in the Extracorporeal Life Support Organization guidelines. However, the optimal targeted temperature for these patients has not yet been adequately investigated. This study aimed to compare the impact of different targeted temperatures on the outcomes in OHCA patients receiving ECMO.

**Methods:**

This was a retrospective analysis of data from the Japanese Association for Acute Medicine (JAAM)-OHCA Registry, a multicentre nationwide prospective database in Japan in which 103 institutions providing emergency care participated. OHCA patients aged ≥ 18 years who required ECMO with TTM between June 2014 and December 2019 were included in our analysis. The primary outcome was 30-day survival with favourable neurological outcomes, defined as a Glasgow–Pittsburgh cerebral performance category score of 1 or 2. Patients were divided into two groups according to their targeted temperature: normothermic TTM (n-TTM) (35–36 °C) and hypothermic TTM (h-TTM) (32–34 °C). We compared the outcomes between the two targeted temperature groups using multivariable logistic regression and inverse probability weighting (IPW).

**Results:**

A total of 890 adult OHCA patients who received ECMO and TTM were eligible for our analysis. Of these patients, 249 (28%) and 641 (72%) were treated with n-TTM and h-TTM, respectively. The proportions of patients with 30-day favourable neurological outcomes were 16.5% (41/249) and 15.9% (102/641), in the n-TTM and h-TTM groups, respectively. No difference in neurological outcomes was observed in the multiple regression analysis [adjusted odds ratio 0.91, 95% confidence interval (CI) 0.58–1.43], and the result was constant in the IPW (odds ratio 1.01, 95% CI 0.67–1.54).

**Conclusion:**

No difference was observed between n-TTM and h-TTM in OHCA patients receiving TTM with ECMO. The current understanding that changes to the targeted temperature have little impact on the outcome of patients may remain true regardless of ECMO use.

**Supplementary Information:**

The online version contains supplementary material available at 10.1186/s13054-022-04256-x.

## Background

Targeted temperature management (TTM) is recommended for patients with out-of-hospital cardiac arrest (OHCA) who remain comatose after the return of spontaneous circulation (ROSC) [1, 2]. In the setting of extracorporeal membrane oxygenation (ECMO) for OHCA, TTM was also associated with good neurological outcomes and is recommended in the Extracorporeal Life Support Organization guidelines [3–7]. Attempts at improving cardiac arrest care have seen an increase in the use of ECMO, which, when combined with TTM, is an intensive care strategy requiring a large investment in logistics [8]. Thus, there is a clear need to investigate the optimal TTM strategy for patients receiving ECMO.

Several specific considerations are required to determine an appropriate target for these patients. As ECMO provides circulatory support as well as tight temperature control, a lower targeted temperature can be achieved easily and applied to the most severe cases of post-ROSC cardiac instability. On the other hand, major complications associated with lower targeted temperatures, such as bleeding and infection, which generally have little impact on the outcome of patients [9, 10], can be critical for the effective functioning of the ECMO circuit. As such, there may be interactions between targeted temperature and the use of ECMO. However, only a few studies with a relatively small number of patients have focused on this issue and have focused on secondary or additional analysis [5, 11–14]. Therefore, the optimal targeted temperature for OHCA patients receiving ECMO has not been adequately addressed.

The aim of this study was to compare the impact of different targeted temperatures on the favourable neurological outcome of OHCA patients receiving ECMO using the database of the Japanese Association for Acute Medicine (JAAM)-OHCA Registry, a multicentre prospective registry.

## Methods

### Design, setting, and patient selection

This was a retrospective analysis of data from the JAAM-OHCA registry, a Japanese multicentre nationwide prospective database that includes pre- and in-hospital information and outcomes among OHCA patients transported to the emergency departments of participating institutions. The ongoing registry started in June 2014 and currently has no anticipated end date. It includes 103 institutions, 79 university hospitals and/or critical care medical centres (CCMC), and 24 community hospitals providing emergency care in each community. The registry includes all OHCA cases that required resuscitation by emergency medical services (EMS) and they were transported to the participating institutions. Patients who were transferred from non-participating institutions were not resuscitated by a physician after hospital arrival, and those refused to be registered were excluded from the registry. The study protocol was approved by the institutional review board of each participating hospital.

For our analysis, OHCA patients aged ≥ 18 years who required TTM with ECMO between June 2014 and December 2019 were included. Patients with unknown targeted temperatures were excluded.

### Data collection

The details have been previously described [15]. Briefly, EMS collected prehospital data according to the international Utstein style [16], in-hospital data were collected by the medical staff of each institution in accordance with a standardized format in an Internet-based system, and the pre- and in-hospital information was integrated by the JAAM-OHCA registry committee.

For our analysis, the following data were collected: patient age, sex, cause of arrest (cardiac or not), presence of a bystander who witnessed the collapse of patient, presence of bystander cardiopulmonary resuscitation (CPR), use of public accessed automated external defibrillator (AED), initially documented rhythm at the scene (shockable, non-shockable or unknown), prehospital epinephrine administration, prehospital advanced airway management, time from EMS call to contact with the patients, time from contact with the patients to hospital arrival, the type of the centre (CCMC or not), the annual number of OHCA patients treated with ECMO at the centre during 1 January to 31 December 2019 (0–8 as low, 9–16 as middle, or 18–35 as high; first, second or third tertile, respectively), initially documented rhythm at the hospital (shockable, non-shockable or presence of pulse), timing of ECMO induction (before or after first ROSC), success of percutaneous coronary intervention (PCI), time from hospital arrival to induction of ECMO, targeted temperature during TTM (32–34 °C as hypothermic-TTM (h-TTM) or 35–36 °C as normothermic-TTM (n-TTM)) [1, 2], and the time from initiation of TTM to achievement of the targeted temperature. The choice of TTM and ECMO protocols was entirely entrusted to the physicians who treated the patients.

### Outcome

The primary outcome was survival 30 days after cardiac arrest with good neurological outcome. This was evaluated using the Glasgow-Pittsburgh cerebral performance category (CPC) scale [17], with categories 1 and 2 used as good neurological outcome. Category 1 indicated good cerebral performance; category 2: moderate cerebral disability; category 3: severe cerebral disability; category 4: coma, or vegetative state; and category 5: death/brain death.

Other collected outcome measures included 30-day survival, completion of TTM (including those who discontinued TTM because of obvious recovery), and complications of TTM (bleeding, arrhythmia, infection, and hypotension-related TTM that needed adjunctive intervention).

### Statistical analysis

We investigated the difference in efficacy between n-TTM and h-TTM combined with ECMO for the management of OHCA. Baseline patient characteristics and outcomes were evaluated using the Mann–Whitney U test or Student’s t test for continuous variables in accordance with normality checked with the Kolmogorov–Smirnov test and Fisher’s exact test for categorical variables.

To investigate the impact of the targeted temperature on each outcome, crude odds ratios (ORs), adjusted odds ratios (AORs), and their 95% confidence intervals (CIs) were calculated using univariable and multivariable logistic regression analyses. Based on previous studies [18–20], we adjusted for preliminary selected factors that were biologically essential and considered to be associated with clinical outcomes, including age, sex, cause of arrest (cardiac or non-cardiac aetiology), bystander witness (yes or no), bystander CPR (yes or no), use of public-access AEDs (yes or no), prehospital epinephrine administration (yes or no), prehospital advanced airway management (yes or no), time from EMS call to contact with the patients, time from EMS contact with the patients to hospital arrival, type of centre (CCMC or non-CCMC), the annual volume of ECMO used for OHCA at each centre, success of PCI, timing of ECMO start (before or after first ROSC), time from hospital arrival to induction of ECMO, and TTM induction time.

We also used propensity score analysis to reduce the effects of confounding factors which accounted for the non-randomized selection of each targeted temperature. We performed inverse probability weighting (IPW) based on the propensity scores. The propensity scores were estimated by using the logistic regression model with all covariates collected in this study other than “TTM induction time” as explanatory variables. The propensity score modelling was assessed by a standardized mean difference (SMD) below 0.10. We used a sandwich variance estimator to estimate the variance for the IPW estimates calculated by the logistic regression model. We also performed a preset subgroup analysis for patients aged ≥ 65 years, with or without witness, and with initial shockable or non-shockable patients.

All P values were two-sided, and the level of significance was set at 0.05. All statistical analyses were performed using R (The R Foundation for Statistical Computing, version 4.12, Saitama, Japan) and EZR (Saitama Medical Center, Jichi Medical University, version 1.55, Saitama, Japan), graphical user interfaces for R [21].

## Results

**Fig. 1:**
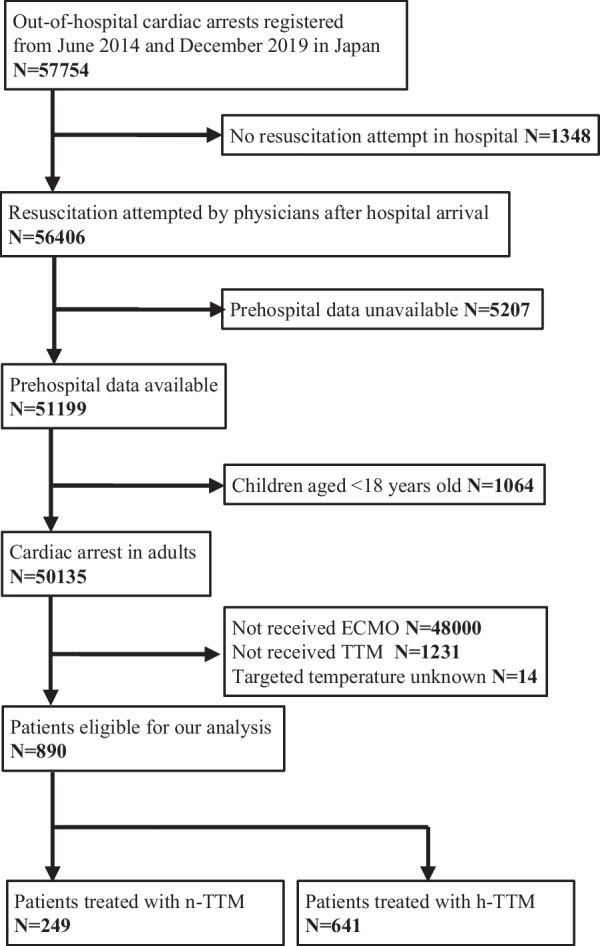
Study flow chart. *ECMO* extracorporeal membrane oxygenation, *TTM* targeted temperature management, *n-TTM* normothermic TTM, *h-TTM* hypothermic TTM

Between June 2014 and December 2019, 57,754 patients were registered in the JAAM-OHCA registry. After excluding 1348 patients, in whom resuscitation was not attempted by physicians, 5207 patients whose prehospital data were unavailable, 1064 patients under 18 years old, 48,000 patients who did not receive ECMO, 1231 patients who did not receive TTM, 14 patients whose targeted temperature was unknown, a total of 890 adult OHCA patients who received ECMO and TTM were eligible for our analysis (Fig. 1).

### Baseline characteristics of study participants

Of the 890 patients, 249 (28%) and 641 (72%) were treated with n-TTM and h-TTM, respectively. Table 1 presents the baseline characteristics of the original cohort and weighted cohort with IPW according to the targeted temperature. No differences in age, witness status, bystander CPR, use of AED, and time-related variables were observed. However, patients with a cardiac cause of arrest [88.4% (22/249) vs 92.7% (594/641)], shockable rhythm [56.2% (140/249) vs 69.7% (447/641)], and a longer TTM induction time [60 (5–193) min vs 110 (31–240) min] and those treated at centres with lower annual usage of ECMO were more likely to be treated with h-TTM than with n-TTM. After adjustment with IPW, no differences in baseline characteristics were observed between the two groups. The distribution of each covariate included in the IPW was well balanced, as all SMDs were < 0.10.

**Table 1:** Baseline characteristics of the study population according to targeted temperature

	Original cohort, *N*, (%)					After adjustment of IPW, %		
	All patients *N* = 890	Missing	n-TTM *N* = 249	h-TTM *N* = 641	SMD	n-TTM	h-TTM	SMD
Basic information								
Men	736 (82.7)	0 (0.0)	207 (83.1)	529 (82.5)	0.016	83.9	82.7	0.032
Age, y, median (IQR)	59 (48–68)	0 (0.0)	60 (50–68)	58 (48–67)	0.115	59 (50–67)	59 (48–68)	0.004
Cardiac cause of arrest	816 (91.5)	0 (0.0)	220 (88.4)	594 (92.7)	0.148	91.8	91.5	0.009
Prehospital information								
Bystander witness	716 (80.5)	0 (0.0)	205 (82.3)	511 (79.7)	0.067	79.3	80.2	0.023
Bystander CPR	439 (49.3)	0 (0.0)	122 (49.0)	317 (49.5)	0.009	48.2	49.3	0.021
Use of public-access AEDs	86 (9.7)	0 (0.0)	22 (8.8)	64 (10.0)	0.039	9.9	9.5	0.013
Initially documented rhythm at the scene		0 (0.0)			0.286			0.006
Shockable rhythm	587 (65.9)		140 (56.2)	447 (69.7)		65.4	65.7	
Ventricular fibrillation	583 (65.5)		139 (55.8)	444 (69.3)				
Pulseless ventricular tachycardia	4 (0.5)		1 (0.4)	3 (0.5)				
Non-shockable rhythm	220 (24.7)		77 (30.9)	143 (22.3)		25.2	24.9	
Pulseless electric activity	141 (15.8)		51 (20.5)	90 (14.0)				
Asystole	79 (8.9)		26 (10.4)	53 (8.3)				
Unknown	83 (9.3)		32 (12.9)	51 (8.0)		9.5	9.4	
Epinephrine	309 (34.7)	0 (0.0)	83 (33.3)	226 (35.3)	0.041	34.8	34.9	0.001
Advanced airway management	803 (90.2)	0 (0.0)	229 (92.0)	574 (89.5)	0.084	89.6	90.1	0.017
EMS call to contact with a patient, min, median (IQR)	7 (5–8)	0 (0.0)	7 (6–8)	7 (5–8)	0.047	7 (5–8)	7 (5–8)	0.005
EMS contact with a patient to hospital, min, median (IQR)^a^	23 (18–30)	10 (1.1)	24 (18–30)	23 (18–30)	0.021	23 (18–30)	23 (18–30)	0.006
Hospital characteristics								
CCMC	838 (94.2)	0 (0.0)	241 (96.8)	597 (93.1)	0.171	92.6	94.2	0.067
Annual number of patients treated with ECMO		0 (0.0)			0.293			0.011
Low volume	319 (35.8)		81 (32.5)	238 (37.1)		36.2	35.9	
Middle volume	313 (35.1)		72 (28.9)	241 (37.6)		35.0	34.9	
High volume	258 (29.0)		96 (38.6)	162 (25.3)		28.7	29.2	
In-hospital information								
Initially documented rhythm at the hospital		0 (0.0)			0.161			0.025
Shockable rhythm	422 (47.4)		110 (44.2)	312 (48.7)		46.3	47.4	
Ventricular fibrillation	407 (45.7)		105 (42.2)	302 (47.1)				
Pulseless ventricular tachycardia	15 (1.7)		5 (2.0)	10 (1.6)				
Non-shockable rhythm	411 (46.2)		116 (46.6)	295 (46.0)		47.9	46.7	
Pulseless electric activity	248 (27.9)		67 (26.9)	181 (28.2)				
Asystole	163 (18.3)		49 (19.7)	114 (17.8)				
Presence of pulse	57 (6.4)		23 (9.2)	34 (5.3)		5.8	5.9	
ECMO induction before first ROSC	596 (67.0)	0 (0.0)	169 (67.9)	427 (66.6)	0.027	66.1	67.1	0.021
Performance of coronary angiography	792 (89.0)	0 (0.0)	210 (84.3)	582 (90.8)				
Success of percutaneous coronary intervention	416 (46.7)	0 (0.0)	116 (46.6)	300 (46.8)	0.004	46.2	46.9	0.013
Hospital arrival to ECMO, min, median (IQR)^a^	29 (20–41)	7 (0.8)	29 (20–45)	29 (20–40)	0.017	29 (20–47)	30 (20–40)	0.023
TTM induction time, min, median (IQR)^a^	95 (28–238)	81 (9.1)	60 (5–193)	110 (31–240)	0.174			

Values are expressed numbers (percentages) unless indicated otherwise

*IQR* interquartile range, *CPR* cardiopulmonary resuscitation, *AED* automated external defibrillator, *EMS* emergency medical service, *CCMC* critical care medical centre, *ECMO* extracorporeal membrane oxygenation, *TTM* targeted temperature management, *N-TTM* normothermic TTM, *H-TTM* hypothermic TTM, *SMD* standardized mean difference, *IPW* inverse probability weighting

^a^Calculated for patients for whom data were available

^b^Comparisons between the two groups were made with Mann–Whitney U test for numeric variables and Fisher's exact test for categorical variables

### Outcomes

Table 2 presents the main outcomes of the study population according to the targeted temperature. Among the n-TTM and h-TTM groups, the proportion of patients with 30-day favourable neurological outcomes, 30-day survival, and completion of TTM was 16.5% vs. 15.9%, 35.3% vs. 35.9%, 65.5% vs. 62.9% in the original cohort; 15.3% vs. 15.4%, 34.3% vs. 35.5%, and 67.0% vs. 62.2% after IPW, respectively.

**Table 2:** Outcomes of the study population according to targeted temperature

	All patients	Original cohort, *N*, %		Crude analysis OR (95% CI)	Multivariable analysis AOR (95% CI)^a^	After adjustment of IPW, %		OR (95% CI)^b^
		n-TTM	h-TTM			n-TTM	h-TTM	
	*N* = 890	*N* = 249	*N* = 641					
30-Day neurological favourable outcome	143 (16.1)	41 (16.5)	102 (15.9)	0.96 (0.65–1.43)	0.91 (0.58–1.43)	15.3	15.4	1.01 (0.67–1.54)
30-Day survival	318 (35.7)	88 (35.3)	230 (35.9)	1.02 (0.75–1.39)	1.00 (0.71–1.41)	34.3	35.5	1.05 (0.76–1.46)
Completion of TTM	566 (63.6)	163 (65.5)	403 (62.9)	0.89 (0.66–1.21)	0.81 (0.57–1.16)	67.0	62.2	0.81 (0.58–1.13)

Values are expressed numbers (percentages) unless indicated otherwise

*TTM* targeted temperature management, *n-TTM* normothermic TTM, *h-TTM* hypothermic TTM, *OR* odds ratio, *AOR* adjusted odds ratio, *CI* confidence interval, *IPW* inverse probability weighting

^a^Shown is the AOR from the multivariable logistic regression analysis adjusted for age, sex, cause of arrest, bystander witness, bystander CPR, use of public-access AEDs, prehospital epinephrine administration, prehospital advanced airway management, time from EMS call to contact with the patients, time from EMS contact with the patients to hospital arrival, type of centre, the annual volume of ECMO used for OHCA at each centre, success of PCI, timing of ECMO start, time from hospital arrival to induction of ECMO, and TTM induction time

^b^Shown is the odds ratio from the univariable logistic regression analysis with IPW

In the multivariable logistic regression analysis, no difference in neurological outcome (AOR 0.91, 95% CI 0.58–1.43), survival (AOR 1.00, 95% CI 0.71–1.41), and completion of TTM (AOR 0.81, 95% CI 0.57–1.16) was observed. This was consistent in the IPW, with neurological outcome (OR 1.01, 95% CI 0.67–1.54), survival (OR 1.05, 95% CI 0.76–1.46), and completion of TTM (OR 0.81, 95% CI 0.58–1.13).

Table 3 presents the occurrence of adverse events among the study population (data calculated using available data). No significant differences were observed between the two targeted temperatures.

**Table 3:** Adverse event of TTM among the study population according to targeted temperature

	All patients	Original cohort		Crude analysis OR (95% CI)	Multivariable analysis AOR (95% CI)^a^
		n-TTM	h-TTM		
	*N* = 617	*N* = 151	*N* = 466		
Any adverse event of TTM	124 (20.1)	29 (19.2)	95 (20.4)	1.08 (0.68–1.78)	1.08 (0.63–1.82)
Bleeding	70 (11.4)	12 (7.9)	58 (12.4)	1.65 (0.86–3.16)	1.47 (0.69–3.10)
Arrhythmia	31 (5.2)	7 (4.6)	24 (5.2)	1.12 (0.47–2.65)	1.28 (0.50–3.31)
Hypotension	76 (12.3)	20 (13.2)	56 (12.0)	0.90 (0.52–1.55)	1.04 (0.55–1.95)
Infection	24 (3.9)	2 (1.3)	22 (4.7)	3.69 (0.86–15.9)	6.09 (0.79–46.8)
Other	8 (1.3)	5 (3.3)	3 (0.6)	0.19 (0.04–0.80)	0.08 (0.01–0.67)

Shown data are calculated for patients for whom data were available (*n* = 617/890)

Values are expressed numbers (percentages) unless indicated otherwise

*TTM* targeted temperature management, *n-TTM* normothermic TTM, *h-TTM* hypothermic TTM, *OR* odds ratio, *AOR* adjusted odds ratio, *CI* confidence interval

^a^Shown is the AOR from the multivariable logistic regression analysis adjusted for age, sex, cause of arrest, bystander witness, bystander CPR, use of public-access AEDs, prehospital epinephrine administration, prehospital advanced airway management, time from EMS call to contact with the patients, time from EMS contact with the patients to hospital arrival, type of centre, the annual volume of ECMO used for OHCA at each centre, success of PCI, timing of ECMO start, time from hospital arrival to induction of ECMO, and TTM induction time

In subgroup analysis, no significant differences or interactions were observed between n-TTM and h-TTM among the subgroups (Table 4).

**Table 4:** 30-day neurological favourable outcome in the subgroups according to targeted temperature

	All patients	Targeted temperature		Crude analysis OR (95% CI)	*P* for interaction
		n-TTM	h-TTM		
	*N* = 890	*N* = 249	*N* = 641		
Age					
≥ 65	30/314 (9.6)	6/99 (6.1)	24/215 (11.2)	1.95 (0.77–4.93)	0.065
< 65	113/576 (19.6)	35/150 (23.3)	78/426 (18.3)	0.74 (0.47–1.16)	
Bystander witness					
Yes	121/716 (16.9)	35/205 (17.1)	86/511 (16.8)	0.98 (0.63–1.51)	0.857
No	22/174 (12.6)	6/44 (13.6)	16/130 (12.3)	0.89 (0.33–2.43)	
Initial rhythm					
Shockable	109/587 (18.6)	25/140 (17.9)	84/447 (18.8)	1.06 (0.65–1.74)	0.127
Non-shockable	17/220 (7.7)	9/77 (11.7)	8/143 (5.6)	0.45 (0.17–1.21)	

Values are expressed numbers (percentages) unless indicated otherwise

*OR* odds ratio, *AOR* adjusted odds ratio, *CI* confidence interval

## Discussion

In this study, we evaluated the impact of targeted temperature on the outcomes of OHCA treated with TTM and ECMO using JAAM-OHCA nationwide registry. There was no difference between n-TTM and h-TTM in terms of impact on favourable neurological outcomes, and this result was constant in the subgroups. There were also no differences in terms of survival, completion of TTM, or TTM complications between the two targeted temperature groups.

At present, few studies with a relatively small number of patients have focused on the targeted temperature among OHCA patients receiving ECMO, and the results are conflicting. Subgroup analyses of two well-designed randomized controlled trials (RCT) in which about half of the participants were cardiac arrest patients, showing consistency with our trial [11, 12]. However, the trials were originally focused on the survival rate of patients with cardiogenic shock and the primary outcome did not include neurological status. On the other hand, a sub-analysis in a systematic review, which did not include the above RCT, showed the superiority of h-TTM over n-TTM [4]. However, this analysis included only 152 patients treated with h-TTM from five observational studies that were not originally used to investigate the impact of targeted temperature [13, 14, 22–24]. Additionally, these studies varied widely in terms of baseline characteristics of study participants and study designs. Thus, it may be less credible when the results from these studies are integrated or generalized because of the bias implicated by the highly heterogeneous study design and baseline data.

In this study, we focused on the neurological outcome. Using a sufficiently large database, which included 890 consecutive OHCA patients treated with ECMO and TTM, multiple logistic regression and IPW were performed to reduce the effects of confounding factors resulting from the non-randomized selection of each targeted temperature. Therefore, we believe that our study partly bridges the knowledge gap and supports the extension of the current understanding that targeted temperature has little impact on patient outcomes in this group of patients.

Notably, the induction time of TTM was significantly shorter in the n-TTM group. Induction time can be of concern provided that the protective effect from the oxidative injury itself is not proportional to the degree of hypothermia in accordance with a previous TTM trial without ECMO [11, 25–27]. However, the induction time in this study (60 min vs. 110 min in the n-TTM and h-TTM group, respectively) was far shorter than those reported in previous studies that suggested a potential benefit of early induction of TTM [28–30], and the induction time differences may be small enough to negate the benefit of early induction [31]. It is also worth mentioning that the occurrence of hypotension due to TTM was similar between the two targeted temperature groups. While a previous large observational study showed that discontinuation of TTM due to hypotension was significantly higher with h-TTM [32], we observed no significant difference in the completion rate of TTM in this study. The circulatory support of ECMO may enable easier implementation of h-TTM. Also, tight temperature control of ECMO potentially limits large increases past the upper threshold temperature of 37.8 °C likely to occur during n-TTM. As such, ECMO may offset the adverse effects of specific targeted temperatures.

In our study, no difference was observed in TTM complications between the two targeted temperature groups, which is consistent with previous trials [26, 31–34]. However, the occurrence rate of each complication showed significant heterogeneity compared with previous studies. Since our study collected “adverse events related to TTM”, underestimation of events which are common among post-cardiac arrest patients or ECMO patients regardless of the use of TTM, such as infection and cannulation site bleeding, could exist. As previous trials defined infection when several criteria, such as new chest X-ray infiltration, leucocytosis, or positive cultures, are fulfilled regardless of the causal relationship to TTM, a much higher occurrence rate of infection may be reported [26, 32]. The rate of bleeding was also higher in these trials with ECMO, probably due to the inclusion of cannulation site bleeding as a complication of TTM [31, 33]. In contrast, the bleeding rate in studies without ECMO was slightly lower, which may be due to the lower use of anticoagulants compared to our patients [26, 32, 34]. As such, our results as well as those of previous trials should be interpreted with caution in terms of complications.

Our study had several limitations. First, patients with fever prevention strategies were lacking because the TTM implementation strategy in this study was based on previous guidelines [35]. As fever prevention strategies and a higher targeted temperature of 37 °C may be selected based on recent practice, further study is needed to clarify the effect of these strategies. Second, while brain death is common among OHCA patients receiving ECMO [36], cessation of mechanical support is not generally accepted in Japan, and early cessation of cost-intensive care owing to socioeconomic problems is rare because of the Japanese insurance system [37, 38]. Based on this, when accounting for the prolonged intensive care in our trial, generalizability in terms of survival rate should be interpreted with caution. However, this may have little impact on neurological outcomes. Third, there was a substantial amount of missing data regarding complications related to TTM, and data on ECMO-specific complications were not collected. As discussed in the previous section, the safety of TTM in ECMO patients requires further investigation. Fourth, our study excluded 1271 ECMO patients without TTM. A certain proportion of these patients might have unintentionally received good temperature control due to the heat exchanger within the ECMO circuit, and this unintentional temperature control may have a similar effect as TTM [39]. However, the outcomes of the patients excluded from our study were extremely worse (Additional file 1: table S1), suggesting a highly heterogeneous population in this group. Furthermore, this registry did not have any data about how their temperatures were controlled, whether they were tolerable for the temperature control, and whether they needed temperature control. Therefore, further study is warranted for this relatively large number of patients, lacking the regarding temperature control. Fifth, since this is an observational study, there could be unmeasured possible confounding factors such as patients' past medical history. RCT may be needed for further validation of our results.

## Conclusion

No difference was observed in the impact of n-TTM and h-TTM on the outcome of OHCA patients receiving ECMO. The current understanding that the targeted temperature has little impact on the outcome of patients may be constant regardless of ECMO use.

## Supplementary Information

**Additional file 1: Table S1.** Outcomes of the patients including those who did not receive TTM.

## Data Availability

Please contact the authors for data requests.

## Abbreviations

TTM: Targeted temperature management
ROSC: Return of spontaneous circulation
OHCA: Out-of-hospital cardiac arrest
ECMO: Extracorporeal membrane oxygenation
JAAM-OHCA registry: Japanese Association for Acute Medicine OHCA registry
CCMC: Critical care medical centres
EMS: Emergency medical service
CPR: Cardiopulmonary resuscitation
AED: Automated external defibrillator
PCI: Percutaneous coronary intervention
h-TTM: Hypothermic TTM
n-TTM: Normothermic TTM
CPC: Glasgow–Pittsburgh cerebral performance category
OR: Odds ratio
AOR: Adjusted odds ratio
CI: Confidence interval
IPW: Inverse probability weighting
SD: Standardized difference
RCT: Randomized controlled trial
