# Supplementary material for: The impact of different targeted temperatures on out-of-hospital cardiac arrest outcomes in patients receiving extracorporeal membrane oxygenation: a nationwide cohort study
Source: Crit Care. 2022 Dec 8;26:380. doi: 10.1186/s13054-022-04256-x (PMC9733046; doi:10.1186/s13054-022-04256-x)
Supplement: Supplementary file 1 — Additional file 1: Table S1. Outcomes of the patients including those who did not receive TTM. [file 13054_2022_4256_MOESM1_ESM.docx]

| **Table S1. Outcomes of the patients including who did not receive TTM** | | | | |
| --- | --- | --- | --- | --- |
|  | All patients | n-TTM | h-TTM | No-TTM |
|  | N=2121 | N=249 | N=641 | N=1231 |
| 30-day neurological favourable outcome | 143 (16.1) | 41 (16.5) | 102 (15.9) | 93 (7.6) |
| 30-day survival | 318 (35.7) | 88 (35.3) | 230 (35.9) | 175 (14.2) |
| Values are expressed numbers (percentages) unless indicated otherwise. TTM, targeted temperature management; n-TTM, normothermic TTM; h-TTM, hypothermic TTM; No-TTM, no intentional temperature control. | | | | |
